# The impact of MRI‐based blood‐brain‐barrier water exchange rate on change in white matter hyperintensities and free water over time

**DOI:** 10.1002/alz70856_099488

**Published:** 2025-12-24

**Authors:** Colleen Pappas, Christopher E. Bauer, Valentinos Zachariou, Xingfeng Shao, Danny JJ Wang, Brian T Gold

**Affiliations:** ^1^ University of Kentucky, Lexington, KY, USA; ^2^ University of Southern California, Los Angeles, CA, USA; ^3^ Sanders‐Brown Center on Aging, Lexington, KY, USA

## Abstract

**Background:**

Understanding biological mechanisms related to cerebrovascular disease (CVD) can provide insight into vascular contributions to cognitive impairment and dementia (VCID). There has been increased interest in exploring blood brain barrier (BBB) function as it relates to other well‐established CVD markers, such as magnetic resonance imaging (MRI) based white matter hyperintensities (WMHs) and free water (FW). Here, we examine how an MRI‐metric of BBB function [water exchange rate (k_w_)] relates to change in WMHs and FW after approximately 2.5 years of follow‐up.

**Method:**

A total of 68 older adults recruited from the University of Kentucky had neuroimaging data. Water exchange rate (k_w_) across the BBB was measured by diffusion prepared pseudo‐continuous arterial spin labeling (DP‐pCASL) at baseline for the whole brain. Cerebral blood flow (CBF) and arterial transit time (ATT) were also measured from the DP‐pCASL sequence. Whole brain WMH volume was quantified using T2‐weighted fluid‐attenuated inversion recovery (FLAIR) and whole brain white matter FW was quantified using diffusion weighted imaging at baseline and follow‐up. Mixed‐effects models controlling for age, gender, and intracranial volume were used to test associations between baseline DP‐pCASL measures (i.e. k_w_, CBF, ATT) and change in WMHs or FW between two visits.

**Result:**

Whole brain k_w_ was not related to WMH volume at baseline, however, higher k_w_ was associated with an increase in WMHs over time. Conversely, higher whole brain CBF was associated with fewer WMHs at baseline, but not with change over time. ATT was not related to WMHs at baseline or over time. When considering the FW measure, higher whole brain k_w_ was related to lower FW cross‐sectionally but results did not persist longitudinally. Both whole brain CBF and ATT were not associated with FW at baseline or over time.

**Conclusion:**

Our preliminary results suggest that BBB k_w_ is related to change in WMHs over time and FW at baseline. Greater water exchange across the BBB may contribute to a greater susceptibility to white matter damage. The FW results are consistent with our prior studies correlating k_w_ and FW. More work is needed to determine the effects of BBB efficiency on downstream cerebrovascular health.

